# Curcumin Protects against Cadmium-Induced Vascular Dysfunction, Hypertension and Tissue Cadmium Accumulation in Mice

**DOI:** 10.3390/nu6031194

**Published:** 2014-03-21

**Authors:** Upa Kukongviriyapan, Patchareewan Pannangpetch, Veerapol Kukongviriyapan, Wanida Donpunha, Kwanjit Sompamit, Praphassorn Surawattanawan

**Affiliations:** 1 Department of Physiology, Faculty of Medicine, Khon Kaen University, Khon Kaen 40002, Thailand; 2 Department of Pharmacology, Faculty of Medicine, Khon Kaen University, Khon Kaen 40002, Thailand; E-Mails: patc_pan@kku.ac.th (P.P.); veerapol@kku.ac.th (V.K.); 3 Department of Physical Therapy, Faculty of Associated Medical Science, Khon Kaen University, Khon Kaen 40002, Thailand; E-Mail: wanida_ams@yahoo.com; 4 Faculty of Medicine, Mahasarakham University, Mahasarakham 44000, Thailand; E-Mail: k_kwanjit@yahoo.com; 5 Research and Development Institute, Government Pharmaceutical Organization, Rama 6 Road, Rajatevee, Bangkok 10400, Thailand; E-Mail: surawatt@gmail.com

**Keywords:** cadmium, curcumin, hypertension, oxidative stress, vascular dysfunction

## Abstract

Curcumin from turmeric is commonly used worldwide as a spice and has been demonstrated to possess various biological activities. This study investigated the protective effect of curcumin on a mouse model of cadmium (Cd)—induced hypertension, vascular dysfunction and oxidative stress. Male ICR mice were exposed to Cd (100 mg/L) in drinking water for eight weeks. Curcumin (50 or 100 mg/kg) was intragastrically administered in mice every other day concurrently with Cd. Cd induced hypertension and impaired vascular responses to phenylephrine, acetylcholine and sodium nitroprusside. Curcumin reduced the toxic effects of Cd and protected vascular dysfunction by increasing vascular responsiveness and normalizing the blood pressure levels. The vascular protective effect of curcumin in Cd exposed mice is associated with up-regulation of endothelial nitric oxide synthase (eNOS) protein, restoration of glutathione redox ratio and alleviation of oxidative stress as indicated by decreasing superoxide production in the aortic tissues and reducing plasma malondialdehyde, plasma protein carbonyls, and urinary nitrate/nitrite levels. Curcumin also decreased Cd accumulation in the blood and various organs of Cd-intoxicated mice. These findings suggest that curcumin, due to its antioxidant and chelating properties, is a promising protective agent against hypertension and vascular dysfunction induced by Cd.

## 1. Introduction

Curcumin, the major active component of curcuminoids, is a natural product found in the rhizomes of turmeric (*Curcuma longa* Linn) of the Zingiberaceae family [1]. Curcumin has been used extensively worldwide as a spice and coloring agent in foods and cosmetics. Several studies have demonstrated the beneficial pharmacological effects of curcumin, including antioxidant, anti-tumorgenic, anti-inflammatory, neuroprotective, and cardioprotective properties [2,3,4]. Moreover, curcumin has been shown in various animal models and clinical studies to be safe even at doses as high as 12 g/day [5]. Our recent study demonstrated that curcumin prevented the development of hypertension in l-NAME-induced hypertensive rats [6]. Curcumin also restored vascular function in mice with endotoxaemia-induced by lipopolysaccharide [7]. Moreover, curcumin has been shown to prevent or reduce neurodegenerative disorders from heavy metal poisoning [8]. It is reported that curcumin efficiently scavenges superoxide anion (O_2_^•−^), hydroxyl radical (OH^•^) and nitrogen dioxide against lead and Cd-induced lipid peroxidation in rat brain homogenates [9].

Cd is a toxic heavy metal contaminant in the environment. Deposition of Cd in living organisms is frequently by ingestion and inhalation. Liver is the primary site of metabolism and accumulation/deposition of acute Cd exposure, resulting in hepatic tissues that are more susceptible to hepatic injuries and necrosis [10]. Meanwhile, kidneys are the critical target organ after chronic occupational or environmental exposure to Cd [11]. Since kidneys are major target organs of chronic Cd toxicity, indirect cardiovascular effects could arise secondarily to renal injury [12]. Many epidemiologic studies have suggested that chronic exposure to cadmium increases the risk of hypertension and cardiovascular disease in the general populations [12,13,14,15,16,17], although other studies showed no association [18,19]. In addition, a large number of animal studies have shown that chronic exposure to Cd can lead to elevation in blood pressure [20,21,22,23]. The exact biological mechanisms that link cadmium exposure and hypertension are unclear. However, considerable evidence suggests that the hypertensive effect of Cd exposure results from complex actions on both the vascular endothelium and vascular smooth muscle cells (VSMCs) [24]. One identifiable factor that plays a central role in Cd-induced hepatotoxicity (acute), nephrotoxicity (chronic), and cardiovascular complications in living organisms which has been the focus of much research is the excessive generation of reactive oxygen species (ROS) and reactive nitrogen species (RNS) [25]. Chronic exposure to Cd not only enhances ROS/RNS generation, but also depletes antioxidant levels, resulting in a state of oxidant/antioxidant imbalance.

There has been no evaluation regarding the effect of curcumin against Cd-induced hypertension. Therefore, the current study aimed to investigate whether curcumin could reduce blood pressure and vascular dysfunction, and whether these effects were associated with an alleviation of oxidative stress, in a mouse model of Cd-induced hypertension and vascular dysfunction.

## 2. Experimental Section

### 2.1. Animal Model of Cd-Induced Hypertension and Vascular Dysfunction

Adult male ICR mice weighing 25–30 g were obtained from the Animal Care Unit of the Faculty of Medicine, Khon Kaen University (Khon Kaen, Thailand). All animal experimental treatment protocols were reviewed and approved by the Animal Ethics Committee of Khon Kaen University. The procedures involving the animals and their care conformed to the institutional guidelines and were in compliance with the National Guidelines for the Care and Use of Animals in Biomedical Research.

After an adaptation periods of seven days, the animals were randomly assigned to six groups of 8–10 animals each: group I: control + propylene glycol (PG), group II: control + curcumin (50 mg/kg b.w.), group III: control + curcumin (100 mg/kg b.w.), group IV: Cd + PG; group V; Cd + curcumin (50 mg/kg b.w.), group VI: Cd + curcumin (100 mg/kg b.w.). The control group received deionized water as drinking water whereas the Cd treated group received drinking water containing CdCl_2_ (100 mg/L) continuously for eight weeks. Curcumin was suspended in PG and intragastrically administered to animals on every other day for eight weeks. The doses of curcumin were based on the results of a previous study which showed that they were sufficient to reduce blood pressure in l-NAME hypertensive rats [6]. Using the body surface area as a factor to convert a dose for translation from mouse to human [26], the high dose of curcumin (100 mg/kg) used in this study is approximately a 500 mg dose for a 60 kg person. The dose is recommended for a daily oral supplement in general populations. The concentration of Cd and duration of exposure followed a previously reported protocol [27].

### 2.2. Assessments of Haemodynamic and Arterial Pressure Reactivity

On the last day of experiments, animals were placed in individual metabolic cages for 24 h. Urine samples were collected and then stored at −20 °C until analysis for nitrate/nitrite as NO oxidative products. Following the urine collection, mice were anaesthetized with an intraperitoneal injection of ketamine:xylazine (100:2.5 mg/kg). The right carotid artery was cannulated and connected to a pressure transducer for continuously monitoring arterial blood pressure using Acqknowledge data acquisition (Biopac System Inc., Santa Barbara, CA, USA). Heart rate was determined by the software from the blood pressure tracing. The left jugular vein was cannulated for infusion of vasoactive agents. After obtaining stable baseline measurements, an endothelium-dependent vasodilator, acetylcholine (ACh; 10 nmol/kg), an endothelium-independent vasodilator, sodium nitroprusside (SNP; 10 nmol/kg), and an alpha sympathomimetic agent, phenylephrine (Phe; 0.03 μmol/kg), were randomly infused intravenously, while blood pressure was continuously monitored. Following the drug infusion, blood pressure was allowed to return to the baseline level and stabilize for at least 5 min. Changes in blood pressure were expressed as percentages of control values obtained immediately before the administration of the test substance (baseline). At the end of the experiment, blood samples were collected from the abdominal aorta into tubes containing EDTA for assays of antioxidant and oxidative stress markers. Subsequently, the aorta was excised rapidly from the animal and used for measurement of O_2_^•−^ production. The liver, kidneys and heart of each animal were excised and weighed.

### 2.3. Biochemical Assays

#### 2.3.1. Assays of Lipid Peroxidation and Protein Oxidation

Lipid peroxidation was measured as malondialdehyde (MDA) production formed in the thiobarbituric acid reactive substances in plasma as previously described [28]. Protein oxidation in plasma was assessed by determining carbonyl groups based on the reaction with 2,4-dinitrophenylephrinenylhydrazine as previously described [29]. The plasma protein content was analyzed by Bradford dye binding assay.

#### 2.3.2. Assay of Nitrate and Nitrite

Urine levels of nitrate and nitrite, NO oxidative products, were measured by a previously described method [30]. Nitrate in urine was reduced to nitrite by nitrate reductase, and then the mixture was reacted with Griess solution (4% sulfanilamide in 0.3% *N*-1-nepthylethylenediamine dihydrochloride) and measured with an ELISA plate reader with a filter wavelength of 540 nm. The amount of urinary nitrate/nitrite concentration was expressed as nmol/mg creatinine.

#### 2.3.3. Assay of O_2_^•−^ Production

The production of O_2_^•−^ in mouse aorta was determined by a lucigenin-enhanced chemiluminescence method as described previously [7,31]. The O_2_^•−^ production in aortic tissue was expressed as relative light unit counts/mg dry wt/min.

#### 2.3.4. Assay of Glutathione

Total glutathione (GSH) in the whole blood was assayed [30], and glutathione disulfide (GSSG) was analyzed after treating the blood sample with 1-methyl-2 vinyl-pyridinum trifate (M2VP), a GSH scavenger. Briefly, a 100 μL sample of whole blood was reacted with 10 μL 33 mM M2VP or distilled water, and subsequently treated with 5% cold metaphosphoric acid to precipitate protein. The supernatant was used in the enzymatic coupling assay for GSH by using a spectrophotometer. The redox status was assessed from the ratio of GSH to GSSG.

### 2.4. Western Blot Analysis

Level of eNOS protein in the aorta samples was determined by Western blotting, as previously described [6] with some modifications. In brief, mouse thoracic aortas were homogenized in cell lysis buffer (Cell Signaling Technology, Inc., New England Biolabs Ltd., Ontario, Canada). The homogenized tissues were centrifuged at 12,000 r.p.m. for 30 min and the supernatant stored at −70 °C until further analysis. Aliquots of tissue homogenates were used for protein assay by the Bradford dye-binding method. A total of 30 μg of protein per sample was separated on 10% SDS-PAGE gel by electrophoresis and transferred to polyvinylidene difluoride membranes. The membranes were blocked with 5% nonfat dry milk in Tris buffered solution containing 0.1% Tween-20 (TBST), and then incubated overnight at 4 °C with the primary antibodies. The antibody used was a mouse monoclonal anti-eNOS (dilution 1:1000, BD Biosciences, San Jose, CA, USA). After incubation, the membrane was washed three times in TBST, and incubated with respective horseradish peroxidase-conjugated secondary antibodies for 2 h at room temperature. After successive TBST buffer washes, the blots were incubated in ECL substrate solution (SuperSignal West Pico Chemiluminescent Substrate: Thermo scientific, Rockford, IL, USA). A mouse monoclonal β-actin antibody (dilution 1:3000, Santa Cruz Biotechnology, Indian Gulch, CA, USA) was used as the Western blot loading control. The densities of the specific protein bands were visualized and captured by ImageQuantTM 400 (GE Healthcare, Pittsburgh, PA, USA). The expressions of eNOS proteins were normalized to β-actin expression from the same sample. The data are shown as percent of normal controls.

### 2.5. Assay of Cd Concentration

Samples of blood, heart, liver, and kidneys were digested with HNO_3_ and H_2_O_2_ under pressure in a closed vessel heated by microwaves. Cd concentrations in all samples were determined as previously described [32,33] by using inductively coupled plasma mass spectrometry (ICP-MS) method (Agilent 7500 ICP-MS model, Santa Clara, CA, USA) according to the manufacturer’s recommendation (The Khon Kaen Laboratory of the Central Laboratory Company, Ltd., Khon Kaen, Thailand). The Cd contents were expressed in μg/L and μg/g tissue weight.

### 2.6. Chemicals and Reagents

Curcumin which used in the current study was generously provided by the Government Pharmaceutical Organization, Bangkok, Thailand. Cadmium chloride (CdCl_2_), 5,5 dithio-bis-2-nitrobenzoic acid (DTNB), ethylenediamine tetraacetic acid (EDTA), glutathione (GSH), thiobarbituric acid (TBA), sodium dodecylsulfate (SDS), butylated hydroxyluene (BHT), metaphosphoric acid (MPA), 2,4-dinitrophenylephrinenylhydrazine (DNPH), *N*-1-nepthylethylenediamine dihydrochloride (NED), 1,1,3,3-tetraethoxypropane, sulfanilamide, guanidine, and phenylephrine hydrochloride were purchased from Sigma-Aldrich Pte. Ltd. (Singapore). Nitrate reductase was obtained from Roche Applied Sciences (Mannheim, Germany). Trichloroacetic acid (TCA), 1-methyl-2-vinyl-pyridinum trifate (M2VP), lucigenin, acetylcholine chloride and sodium nitroprusside were obtained from Fluka Chemika Co., Ltd. (Buchs, Switzerland). All other chemicals used were of analytical grade.

### 2.7. Statistical Analysis

Data obtained were expressed as mean ± S.E., and n refers to the number of animals used. The significance of differences between means was analyzed by one-way analysis of variance (ANOVA) and followed by post-hoc Duncan’s multiple range test. Statistical significance was assigned at a *p* value of less than 0.05.

## 3. Results

### 3.1. Effect of Curcumin on Haemodynamic Status and Vascular Reactivity

**Figure 1:**
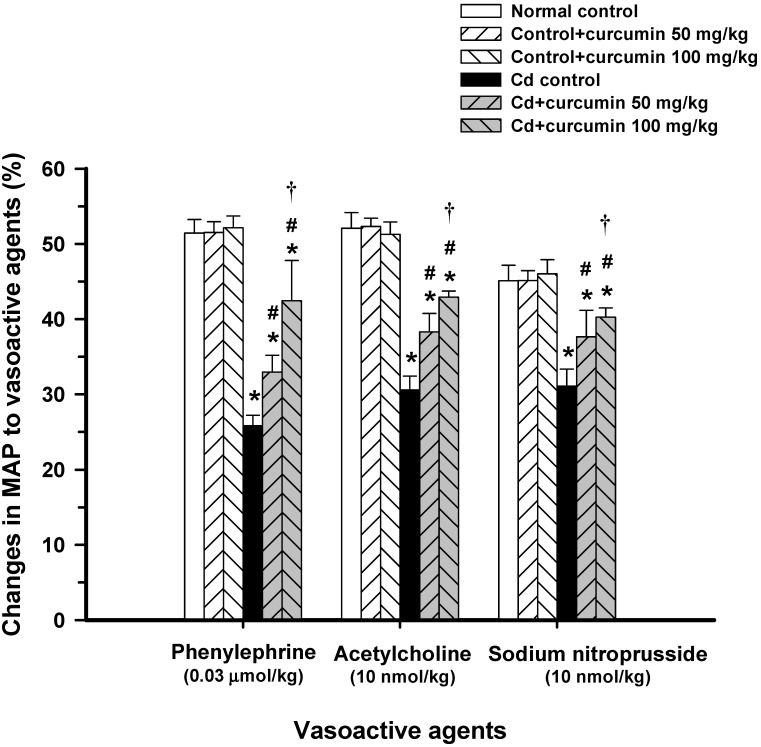
Effects of curcumin co-administration on vascular response to vasoactive agents, phenylephrine, acetylcholine, and sodium nitroprusside. Mice received CdCl_2_ (100 mg/L in drinking water) alone or combined with curcumin (50 or 100 mg/kg, p.o.). MAP, mean arterial pressure. Results are expressed as mean ± S.E., *n* = 8–10/group. * *p* < 0.05 compared with normal control group; **^# ^***p* < 0.05 compared with CdCl_2_ control group, **^† ^***p* < 0.05 compared with Cd + curcumin (50 mg/kg).

Administration of curcumin at a dose of 50 or 100 mg/kg did not alter arterial blood pressure or heart rate in normal control mice. Daily intake of CdCl_2_ at the study concentration caused a significant increase in systolic, diastolic, and mean arterial blood pressure levels when compared with the normal control group (Table 1), whereas there were no changes in the heart rate. Curcumin at tested doses significantly decreased systolic, diastolic and mean arterial blood pressure levels of mice exposed to Cd (*p* < 0.05, Table 1). Moreover, it is found that curcumin co-administration at 100 mg/kg had more profound effects on diastolic and mean arterial blood pressure levels (Table 1). Cd-treated mice that received curcumin (50 or 100 mg/kg) showed a significant reduction in mean arterial blood pressure to approximately 17%–26% of the Cd-treated controls. It was noted that curcumin at high dose (100 mg/kg) was able to maintain blood pressure to near normal values. Importantly, administration with Cd drastically impaired the vascular responses to various vasoactive agents, Phe, ACh, and SNP shown in Figure 1. These results indicate that Cd caused an impairment of vasorelaxation as well as vasoconstriction. Curcumin at high dose significantly restored the response of Phe (42.4% *vs.* 25.8%), ACh (42.9% *vs.* 30.6%) and SNP (40.2% *vs.* 31.1%) when compared to those found in Cd-treated controls (*p* < 0.05, Figure 1). Altogether, curcumin dose-dependently protected against hypertension and prevented impairment of vascular responsiveness to vasoactive agents induced by Cd.

**Table 1:** Effects of curcumin co-administration on cadmium-induced changes in blood pressure levels and heart rates.

Parameters	Normal Control	Normal control + Curcumin (mg/kg)		Cd Control	Cd + Curcumin (mg/kg)	
		50	100		50	100
Systolic pressure (mmHg)	116 ± 3	120 ± 1	122 ± 1	155 ± 1 *	130 ± 1 *^,#^	129 ± 3 *^,#^
Diastolic pressure (mmHg)	82 ± 3	85 ± 1	85 ± 2	113 ± 2 *	102 ± 1 *^,#^	91 ± 4 ^#^^,†^
Mean arterial pressure (mmHg)	96 ± 2	97 ± 2	95 ± 1	136 ± 2 *	113 ± 1 *^,#^	101 ± 4 ^#^^,†^
Heart rate (beats/min)	330 ± 8	337 ± 7	340 ± 5	340 ± 8	331 ± 4	340 ± 7

Mice received CdCl_2_ (100 mg/L in drinking water) alone or combined with curcumin (50 or 100 mg/kg, p.o.). Data are expressed as mean ± S.E., *n* = 8–10/group. * *p* < 0.05 compared with normal control group, **^# ^***p* < 0.05 compared with Cd control group, **^† ^***p* < 0.05 compared with Cd + curcumin (50 mg/kg).

### 3.2. Effect of Curcumin on Oxidant and Antioxidant Status

To evaluate whether the maintenance of blood pressure and vascular responsiveness in Cd-exposed mice was associated with oxidant formation and antioxidant effect of curcumin, we measured the following parameters which are related to oxidative stress and antioxidant redox status, including vascular O_2_^•−^ production, urinary nitrate/nitrite, plasma MDA, plasma protein carbonyls, blood GSH, and the redox ratio of GSH/GSSG.

Curcumin at doses of 50 or 100 mg/kg did not change the normal levels of oxidant and antioxidant parameters in control mice (Table 2). However, chronic Cd intake caused almost 10-fold increase of O_2_^•−^ production in the thoracic aortas and more than 2-fold increase in urinary nitrate/nitrite levels when compared with normal controls (*p* < 0.01, Table 2). These data provide evidence of oxidative stress in mice exposed to Cd. Curcumin at tested doses significantly lowered the rate of O_2_^•−^ production in thoracic aortas and decreased urinary levels of nitrate/nitrite in comparison to normal control values (*p* < 0.01, Table 2).

Regarding the biomarkers of oxidative damage, it was found that plasma MDA and protein carbonyls of Cd-exposed mice were significantly higher than normal control values (*p* < 0.01, Table 2). Interestingly, curcumin, especially at high dose, markedly suppressed the elevation of plasma MDA and protein carbonyls to values comparable with those of normal controls (Table 2). These results indicate that curcumin reduces lipid peroxidation and protein oxidation caused by Cd intoxication. Cellular redox of GSH plays an important role in regulating various redox-sensitive proteins in cellular signaling; therefore, alteration of cellular functions may be due to changes in redox status. Mice-treated with Cd showed a dramatic reduction in blood GSH and redox ratio of GSH/GSSG. Changes in redox status were well correlated with the levels of free radical generation and degrees of lipid peroxidation and protein oxidation (Table 2). Curcumin co-administration with Cd markedly prevented loss of GSH and restored redox status of the blood cells. These results suggest that curcumin decreases the oxidants generated during Cd exposure and increases endogenous antioxidant formation. Interestingly, the alleviation of oxidative stress by curcumin in Cd-intoxicated mice was well correlated with the restoration of blood pressure and vascular responsiveness.

### 3.3. Effect of Curcumin on Vascular eNOS Protein Expression

**Figure 2:**
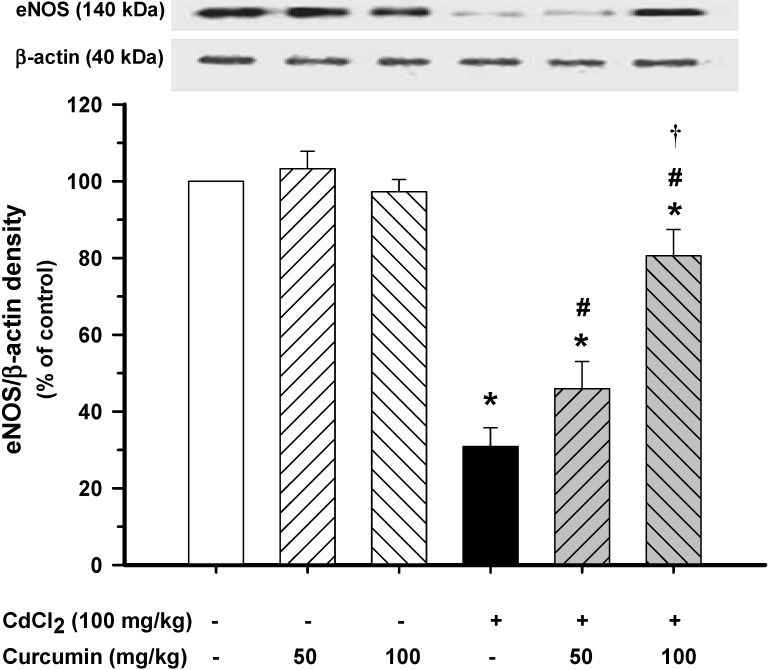
Effects of curcumin co-administration on expression levels of eNOS protein in thoracic aortas. Plots show the densitometric intensities of eNOS protein expressions for each condition, normalized against β-actin expression and presented in percent of normal controls. Results are expressed as mean ± S.E., Each column represents the mean of four experiments. * *p* < 0.05 compared with normal control group; **^# ^***p* < 0.05 compared with Cd control group, **^† ^***p* < 0.05 compared with Cd + curcumin (50 mg/kg).

To investigate whether curcumin decreases blood pressure and improves vascular reactivity through the induction of vascular eNOS, Western blot analysis for eNOS expression was performed. This study showed that eNOS protein expression in the aortas was decreased by 70% in Cd-exposed mice. Consistent with the restoration of haemodynamic status and vascular reactivity, curcumin co-administration, especially at high doses, significantly increased the eNOS protein expression when compared with the Cd control group (*p* < 0.01, Figure 2).

### 3.4. Effect of Curcumin on Cd Concentrations

Data in Table 3 demonstrate the levels of Cd accumulations in various soft tissues, including heart, aorta, liver and kidneys, and in the whole blood of all experimental groups. In the normal control group, the levels of Cd in aorta, liver and kidneys were very low and undetectable in the heart tissues. A marked increase of Cd concentrations was found in the blood and organs of mice after Cd exposure for eight weeks. Curcumin co-administration significantly reduced Cd accumulations in the blood and organs (*p* < 0.01, Table 3).

## 4. Discussion

The present study demonstrates that curcumin protects against hypertension, vascular dysfunction and oxidative stress induced by Cd in mice. These ameliorative effects of curcumin against Cd toxicity might be mediated by its antioxidant and chelating properties.

**Table 2:** Effects of curcumin co-administration on cadmium-induced oxidative stress and changes in redox status.

Parameters	Normal Control	Normal control + Curcumin (mg/kg)		Cd Control	Cd + Curcumin (mg/kg)	
		50	100		50	100
Aortic superoxide anion (Counts/mg dry wt./min)	161.2 ± 15.1	160.4 ± 9.5	158.7 ± 8.6	1202.4 ± 121.9 *	851.9 ± 73.6 *^,#^	711.5 ± 23.0 *^,#^^,†^
Urinary nitrate/nitrite (nmol/mg creatinine)	909.7 ± 74.8	895.5 ± 57.5	920.0 ± 41.7	2074.6 ± 102.4 *	1499.9 ± 43.4 *^,#^	1116.5 ± 156.7 ^#^^,†^
Plasma malondialdehyde (μM)	15.4 ± 0.6	15.0 ± 1.3	15.9 ± 0.4	32.3 ± 2.4 *	25.6 ± 3.3 *^,#^	17.8 ± 1.5 ^#^^,†^
Plasma protein carbonyls (nmol/mg protein)	1.4 ± 0.09	1.4 ± 0.06	1.4 ± 0.06	3.5 ± 0.4 *	2.0 ± 0.2 *^,#^	1.7 ± 0.4 ^#^
Blood GSH (μM)	825 ± 69	811 ± 46	801 ± 31	270 ± 21 *	510 ± 40 *^,#^	603 ± 32 *^,#^^,†^
Blood GSH/GSSG	149 ± 13	143.5 ± 11	146 ± 11	27 ± 3 *	58 ± 7 *^,#^	109 ± 5 *^,#^^,†^

Mice received CdCl_2_ (100 mg/L in drinking water) alone or combined with curcumin (50 or 100 mg/kg, p.o.). GSH, reduced glutathione; GSSG, oxidized glutathione. Data are expressed as mean ± S.E., *n* = 8–10/group. * *p* < 0.01 compared with normal control group, **^# ^***p* < 0.01 compared with Cd control group, **^† ^***p* < 0.05 compared with Cd + curcumin (50 mg/kg).

**Table 3:** Effects of curcumin co-administration on blood and tissue cadmium accumulation levels.

Treatment	Heart (μg/g Tissue)	Aorta (μg/g Tissue)	Liver (μg/g Tissue)	Kidneys (μg/g Tissue)	Whole Blood (μg/L)
Normal control	undetectable	0.023 ± 0.003	0.035 ± 0.006	0.116 ± 0.006	2.20 ± 0.007
Cd control	0.50 ± 0.039	0.24 ± 0.023 *	11.37 ± 1.53 *	19.75 ± 2.25 *	60.40 ± 6.95 *
Cd + Curcumin 50 mg/kg	0.41 ± 0.063	0.22 ± 0.005 *	7.25 ± 0.52 *^,^^#^	15.83 ± 0.81 *^,^^#^	46.46 ± 6.35 *^,^^#^
Cd + Curcumin 100 mg/kg	0.24 ± 0.059 ^#^^,^^†^	0.09 ± 0.004 *^,^^#^^,^^†^	6.87 ± 0.85 *^,^^#^	12.28 ± 0.79 *^,^^#^^,^^†^	23.30 ± 1.59 *^,^^#^^,^^†^

Mice received CdCl_2_ (100 mg/L in drinking water) alone or combined with curcumin (50 or 100 mg/kg, p.o.). Data are expressed as mean ± S.E., *n* = 4–5/group. * *p* < 0.05 compared with normal control group, **^# ^***p* < 0.05 compared with Cd control group, **^† ^***p* < 0.05 compared with Cd + curcumin (50 mg/kg).

Generally, cardiac output and peripheral resistance are the two determinants of arterial pressure. Based on the haemodynamic data of Cd-intoxicated mice, we found that curcumin, especially at high doses, decreased the diastolic blood pressure levels more than systolic blood pressure levels. Meanwhile, there were no changes in the heart rate among all groups. These findings suggest that the preventive effect of curcumin against hypertension during Cd exposure might be due to its vasodilatory effect on the arteries and arterioles, since diastolic pressure relates more closely to the vascular resistance than to the cardiac function [34]. Considerable evidence suggests that the hypertensive effect of Cd exposure results from complex actions on both the vascular endothelial cells and VSMCs [24]. There is strong evidence that Cd decreases the functional availability of the potent vasodilator NO and leads to increase in blood pressure [35]. Consistent with this, the present experiment revealed a significant increase in blood pressure and decrease in vascular reactivity to endothelial-dependent vasodilator, ACh. It has been suggested that the suppression of vascular response to ACh after Cd exposure as frequently denoted as endothelial dysfunction may be due to muscarinic cholinergic and NO-dependent pathway dysfunction in the vascular endothelium [22]. An impairement of Phe-induced contraction found in our study may be related to Cd-inhibited extracellular Ca^2+^-independent contractile response by directly disrupting the intracellular signal transduction pathway(s), probably via a Ca^2+^ independent mechanism [36]. In addition, an attenuation of vascular reactivity to SNP observed in our study might be attributable to long-term damage of VSMCs caused by Cd intoxication. It is reported that Cd promotes the proliferation of VSMCs and enhances the production of extracellular matrix component [37], which thereby increases the arterial stiffness and blood pressure.

It is well documented that oxidative stress plays a major role in vascular dysfunction and also exerts its effects in other pathogenic conditions [38]. The present study has demonstrated that Cd-induced high blood pressure is associated with decreased availability of NO, because the large amount of O_2_^•−^ rapidly reacted with NO to form peroxynitrite (ONOO^−^) [39]. Peroxynitrite is a potent free radical that can switch eNOS, via the oxidation of tetrahydrobiopterin, from a NO-generating to a superoxide-generating enzyme (a process termed NOS uncoupling) [40].

The source of ROS formation may be from the mitochondria, where Cd interacts with reactive thiols in the mitochondrial membrane and causes mitochondrial permeability transition, which inhibits the respiratory chain reaction, and then generates ROS [41]. In our study, we have found that curcumin markedly decreased O_2_^•−^ production in the aorta and reduced urinary nitrate/nitrite levels in a dose-dependent manner, indicating that curcumin reduced the generation of O_2_^•−^ and NO which might decrease ONOO^−^ formation. It seems likely that the antioxidant activity of curcumin is well correlated with the decrease in blood pressure of Cd-exposed mice. Interestingly, curcumin has phenol rings that act as electron traps to scavenge ONOO^−^, O_2_^•−^ and OH^•^. Curcumin also has a diketone group that can react with OH^•^ and H_2_O_2_ and contains two phenyl methoxy groups, which contribute to the suppression of NF-κB activation [42]. A previous study revealed that the diketone groups of curcumin are required for suppression of NF-κB activity and consequently linked to inhibition of expression of iNOS, NADPH oxidase enzymes and other pro-inflammatory cytokines [43].

Decreased eNOS protein expression found in this study was associated with increased blood pressure and impaired ACh-dependent vasorelaxation after Cd exposure. These findings are in agreement with a previous report finding that animals, which received Cd, had a significant decrease in eNOS protein level [22]. Moreover, it has been demonstrated that Cd inhibits NO production in endothelial cells via inhibiting eNOS phosphorylation, which leads to endothelial dysfunction [44]. As discussed earlier, curcumin could suppress O_2_^•^^−^ and increase NO formation, and thus prevents ONOO^−^ production and increases NO bioavailability in this study.

Cd induced a severe suppression of GSH redox ratio with diminuation of GSH or alternatively, Cd may inactivate some GSH synthesis enzymes. We found that Cd reduced the blood GSH and suppressed GSH redox ratio. It has been reported that the disturbance in GSH causes oxidative damage through the deterioration of lipid, protein and DNA resulting in various pathological conditions in humans and animals [45]. This finding is in agreement with the previous study that hepatic lipid peroxidation, hydroperoxide and protein carbonyls content were significantly increased in rats treated with 50 mg/kg CdCl_2_ in their drinking water for four weeks [46]. Curcumin could induce increased GSH, and normalized GSH redox ratios. In an oxidative stress condition, curcumin has been shown to induce the expression of gamma-glutamyl cysteine ligase, the rate limiting enzyme for GSH synthesis, and a downstream gene of the Nrf2-ARE signaling pathway where curcumin is a potent inducer of transcription factor Nrf2 activator [47]. Our data showed the association of improved vascular dysfunction with the increased redox status and decreased oxidative stress after curcumin administration in mice exposed to Cd.

Cd accumulates mainly in the heart, aorta, liver and kidneys and causes severe tissue damage in these organs [21,48]. It is noted that Cd content in the whole kidney of the group that received Cd and a low dose of curcumin in this study was close to the average of Cd accumulation in the kidney of the Swedish donors [49]. A recent study by our group has shown that 2,3-dimercaptosuccinic acid, a known metal chelator, protected cardiovascular function by metal chelation and inhibition of oxidative stress [21]. Interestingly, this study has found that the Cd contents in the heart, aorta, liver, kidneys, and whole blood were decreased in mice receiving Cd and curcumin. Based on the electrochemical studies, it has been suggested that there might be a metal-ligand interaction between Cd and curcumin, thereby reducing the heavy metals load in the body [50], and reducing the toxic effects of Cd. Moreover, it is possible that curcumin might interfere with the gastrointestinal absorption of Cd, thereby causing a reduction in Cd concentration in the blood and tissues. This suggests the potential chelating effect of curcumin. However, curcumin at low and high doses improved haemodynamic and vascular responsiveness to a great extent; particularly low doses of curcumin improved those parameters while Cd levels in the heart and aorta were minimally reduced. For this reason, it seems that the chelating effect of curcumin in our study might have a minor contribution. Nonetheless, further investigation of this issue is needed.

## 5. Conclusions

This study provides evidence concerning the beneficial effects of curcumin on reduced hypertension, improved vascular dysfunction and alleviated oxidative stress in mice after Cd exposure for eight weeks. The possible mechanisms involved in these effects in Cd-exposed mice may be due to an increase in NO bioavailability by up-regulation of eNOS protein and a strong antioxidant activity of curcumin. Collectively, this study suggests the beneficial effect of curcumin as a dietary supplement to prevent Cd-induced hypertension and vascular dysfunction.
